# Caprine leptospirosis: Hematobiochemical and urinalyses studies

**DOI:** 10.14202/vetworld.2016.337-341

**Published:** 2016-03-29

**Authors:** Priti Dilipsinh Vihol, Jignesh M. Patel, Jatin H. Patel, Mahesh C. Prasad, Irsadullakhan H. Kalyani, Balkrushna P. Brahmkshtri

**Affiliations:** 1 Department of Veterinary Pathology, Vanbandhu College of Veterinary Science and Animal Husbandry, Navsari Agricultural University, Navsari - 396 450, Gujarat, India; 2 Department of Veterinary Pharmacology and Toxicology, Vanbandhu College of Veterinary Science and Animal Husbandry, Navsari Agricultural University, Navsari - 396 450, Gujarat, India; 3 Department of Veterinary Microbiology, Vanbandhu College of Veterinary Science and Animal Husbandry, Navsari Agricultural University, Navsari - 396 450, Gujarat, India; 4 Department of Animal Genetics and Breeding, Vanbandhu College of Veterinary Science and Animal Husbandry, Navsari Agricultural University, Navsari - 396 450, Gujarat, India

**Keywords:** goats, hematobiochemical, leptospirosis, urinalysis

## Abstract

**Aim:**

The present study was designed to evaluate clinicopathological alterations in naturally occurring leptospirosis in goats of South Gujarat region, Gujarat.

**Materials and Methods:**

A total 459 blood/serum and 292 urine samples were collected from different districts of South Gujarat region, India. Blood/serum and urine samples were subjected to hematobiochemical analyses and urinalyses. The serum samples were screened for anti-leptospiral antibodies using the microscopic agglutination test (MAT). On the bases of presence or absence of anti-leptospiral antibodies in serum, seropositive and seronegative groups were made. The results were analyzed using standard statistical methods to know pathological changes in the disease.

**Results:**

In MAT, out of 459, 116 goats were seropositive, and 343 were seronegative. In hematobiochemical analyses, statistically significant (p<0.01) decrease in values of packed cell volume, hemoglobin (Hb) concentration, mean corpuscular Hb concentration and total protein and increased activity/level of alanine aminotransferase, aspartate aminotransferase and total bilirubin between seropositive and seronegative goats were noted. Urinalyses did not reveal any specific changes. In the dark field microscopy, urine samples were found to be negative for leptospires.

**Conclusion:**

Hematobiochemical changes noted in seropositive goats were indicative of hepatic damage, and this knowledge would aid in the therapeutic management of the disease.

## Introduction

Leptospirosis, caused by pathogenic spirochetes (i.e. *Leptospira interrogans*), is reported in human and many species of animals. The most common serovars of *L. interrogans* involved in caprine abortion are Grippotyphosa and Pomona. Although sheep are relatively resistant to leptospirosis, goats are susceptible, with abortions occurring at the time of leptospiremia. It runs into acute or chronic course with common symptoms such as anemia, icterus, and hemoglobinemia; others are afebrile and not icteric [1]. Literature is available indicating clinical pathology of disease in many species of animals [2-7].

However, most of these studies were experimental. The disease leptospirosis usually goes unnoticed in small ruminants as most of the time it runs in subclinical form. There is a paucity of information on clinicopathological profiles indicating effects on body/various organs especially in naturally infected/exposed goats in leptospirosis as the disease usually go unnoticed in these animals because of its usual manifestation as subclinical form.

Hence, the present investigation was aimed to study hematology, biochemical profiles, and urinalyses among *Leptospira* infected goats. Microscopic agglutination test (MAT) [8] was used to differentiate infected and uninfected animals. These results were considered as a base to correlate clinical pathology of the disease.

## Materials and Methods

As per CPCSEA guidelines, study involving clinical samples does not require the approval of Institute Animal Ethics Committee and the authors were permitted by animal owners for sampling.

### Collection of blood and serum samples

Blood/serum samples (459) were aseptically collected randomly via jugular vein (K3 ethylenediaminetetraacetic acid) from goats (both sex) reared in villages (417) of various districts (Navsari, Surat, Tapi, Valsad) of South Gujarat and Surat Slaughter House (42). At the time of collection of the sample on the base of history and clinical observation such as pale/icterus mucous membrane, anorexia, fever agalactia, mastitis, or abortion, animals were divided into clinically ailing (n=126) and apparently healthy (n=333) goats. Samples were transported under cold chain system to the pathology laboratory. Whole blood samples were used for hematology. To obtain serum whole blood was kept in slanting position in 9.0 ml plain vacutainers until serum was extracted out. The 9.0 ml plain vacutainers were centrifuged at 7000 rotation a minute (rpm) for 10 min if needed. The straw colored serum was collected into two sets of 1.5 ml sterile cryovials and aliquoted. One set was stored at −20°C for MAT while the other set was used for analyses of biochemical parameters.

### Collection of urine samples

Urine samples (n=292) were collected randomly from the same population of goats during blood collection at the same visit. Midstream urine samples were collected in plastic containers after cleaning the vulva or preputial opening. Sufficient (30-50 ml) amount of urine was collected from each animal. In addition, urine samples were also collected from slaughtered animals (goats-42) directly from the urinary bladder. All the urine samples were transported to the laboratory as soon as possible.

### Hematological analyses

Blood samples were subjected to various hematological analyses, i.e., hemoglobin estimation (Hb), packed cell volume (PCV), total erythrocyte count (TEC), mean corpuscular volume (MCV), mean corpuscular Hb (MCH), MCH concentration (MCHC), and total leukocyte count (TLC) were analyzed using Automatic Whole Blood Analyzer (Medonic CA 620/530 VET, Boule Medical AB, Sweden) while differential leukocyte count (DLC) on Giemsa stained blood smears was performed manually.

### Serum biochemical analyses

Within 24 h of sample collection, serum samples were analyzed for various serum biochemical tests, i.e., total protein (TP), albumin, total bilirubin, aspartate aminotransferase (AST), alanine aminotransferase (ALT), alkaline phosphatase (ALP), creatinine, blood urea nitrogen (BUN) using Randox Kits (M/S Randox Laboratory Limited., 55 Diamond Road, Crumlin, Co. Antrim, BT 29 4 QY, United Kingdom) in Auto Serum Analyzer (M/S Chemwell Awareness Technology, Inc.).

### MAT

All the sera collected were tested for antibodies against live antigens of *Leptospira* sp. (serovars Pyrogenes, Australis, Bankinang, Grippotyphosa, Patoc-1, Pomona, Icterohemorrhagiae, Hebdomadis, Canicola, Hardjo, Ballum, Bataviae, Tarassovi, Shermani, Kaup, Hurstbridge, and Javanica) by MAT at Leptospirosis Reference Laboratory, Government Medical College, Surat [9] and National Institute of Veterinary Epidemiology and Disease Informatics, Indian Council of Agricultural Research, formerly Project Directorate on Animal Disease Monitoring and Surveillance, Hebbal, Bengaluru using standard procedure. Animals were considered seropositive if MAT titer was found above 40 [10,11].

### Urinalyses

Urine samples were subjected to various tests to determine different parameters such as leukocytes, nitrite, urobilinogen, protein, pH, blood, specific gravity, ketone bodies, bilirubin, and glucose using Reagent Strips (10P) (M/S Beacon Diagnostic Pvt. Ltd., Kabilpore, Navsari). In addition, dark field microscopy (DFM) was performed on urine sediment obtained post-filtration (0.45 µm pore size filters, Pall life science) and centrifugation at 7800 rpm for 10 min to detect the presence of *Leptospira* organism, if any.

To investigate attributes of clinical pathology in leptospirosis among goats, related details of seropositive and seronegative goats were compared. So here, MAT was used as screening test to know the status of the animal, and then these animals were grouped into two groups, *viz*., seropositive and seronegative.

### Statistical analysis

Student’s t-test was carried out using Statistical Packages for Social Science software (version 17).

## Results

In MAT, 116/459 samples were seropositive, and the rest (343/459) were seronegative. Among these 116 seropositive animals, 18 (out of 126) were clinically ailing and 98 (out of 333) were apparently healthy. Among seropositive ailing animals, symptoms or histories such as pale/icterus mucous membrane (8/18), anorexia(1/18), fever (4/18), agalactia (1/18), mastitis (1/18), or abortion (3/18). Most of the seropositive animals were apparently healthy, and their MAT titer ranged between 40 and 10,240.

The detail values of various hematological parameters (mean ± standard error) analyzed from seronegative and seropositive goats are presented in Table-1. Except the values of TLC and DLC, all the other values (Hb, PCV, TEC, MCV, MCH, and MCHC) registered a decrease in seropositive groups in comparison to seronegative groups. Moreover, highly significant (p<0.01) decrease in values of PCV, Hb, and MCHC were noted.

**Table-1:** Details of hematological parameters studied in goats.

Parameters studied	Mean±SE	

	Seronegative (n=343)	Seropositive (n=116)
Hb (g/dl)	7.47±0.10	6.53±0.11**
PCV (%)	20.87±0.32	18.40±0.33**
TEC (×10^6^/µl)	8.24±0.14	7.98±0.26^NS^
TLC (×10^3^/µl)	10.14±0.24	10.32±0.39^NS^
MCV (fl)	28.94±0.80	27.10±1.28^NS^
MCH (pg)	10.25±0.26	9.67±0.46^NS^
MCHC (g/dl)	36.14±0.23	35.71±0.29*
DLC (%)		
Neutrophils	35.97±0.82	37.73±1.32^NS^
Lymphocytes	56.20±0.83	14.88±1.38^NS^
Eosinophils	4.43±0.23	4.44±0.41^NS^
Monocytes	2.58±0.05	2.48±0.12^NS^
Basophils	0.82±0.02	0.85±0.03^NS^

** Highly significant at p<0.01 as compared to seronegative animals,

* Significant at p<0.05 as compared to seronegative animals,

^NS^Non-significant at p<0.05 as compared to seronegative animals. n=Number of animals, SE=Standard error, Hb=Hemoglobin, PCV=Packed cell volume, TEC=Total erythrocyte count, TLC=Total leukocyte count, MCV=Mean corpuscular volume, MCH=Mean corpuscular hemoglobin, MCHC=Mean corpuscular hemoglobin concentration, DLC=Differential leukocyte count

As shown in the Table-2, analyses of serum for serum biochemical parameters revealed increased activity/level of ALT, AST, and total bilirubin and decreased the level of TP at a significant level (p<0.01) between seropositive and seronegative goats. The activity/level of parameters such as ALP, BUN, creatinine, and albumin did not differ significantly between seropositive and seronegative groups.

**Table-2:** Various biochemical parameters studied in goats.

Parameters studied	Mean±SE		

	Seronegative (n=343)	Seropositive (n=116)
ALT (IU/L)	25.65±0.46	26.14±0.91**
AST (IU/L)	74.54±1.48	80.20±4.39**
ALP (IU/L)	100.74±4.98	104.89±7.2^NS^
Total bilirubin (mg/dl)	0.54±0.006	0.70±0.015**
BUN (mg/dl)	15.67±0.28	15.71±0.40^NS^
Creatinine (mg/dl)	0.88±0.02	0.89±0.03^NS^
TP (g/dl)	9.68±0.02	7.64±0.26*
Albumin (g/dl)	2.52±0.04	2.11±0.08^NS^

** Highly significant at p<0.01 as compared to seronegative animals,

* Significant at p<0.05 as compared to seronegative animals,

^NS^Non-significant at p<0.05 as compared to seronegative animals. n=Number of animals, SE=Standard error, ALT=Aspartate aminotransferase, ALT=Alanine aminotransferase, ALP=Alkaline phosphatase, BUN=Blood urea nitrogen, TP=Total protein

All the 312 urine samples collected were subjected to DFM to screen presence of leptospires if any, however, leptospires could not be detected in any sample. Detailed result of urinalyses is presented in Table-3. On urinalyses, among seropositive goats (n=42), mean ± SE values of pH and specific gravity were 7.69±0.068 and 1.011±0.008, respectively, in comparison to seronegative goats (n=250): pH 7.73±0.025 and specific gravity 1.012±0.003. The difference between seropositive and seronegative groups in respect of pH and specific gravity did not differ significantly (p<0.05). Other parameters such as leukocytes, nitrite, urobilinogen, red blood cells, ketone bodies, bilirubin, and glucose were found to be negative both in seropositive and seronegative goats.

**Table-3:** Details of urine analyses (mean±SE) in goats.

Parameters studied	Seronegative (n=250)	Seropositive (n=42)
Leukocytes (cells/μL)	Negative	Negative
Nitrate (μmol/L)	Negative	Negative
Urobilinogen (µmol/L)	Negative	Negative
Protein (g/L)	Negative	Negative
pH	7.73±0.025	7.69±0.068^NS^
Blood (cells/µL)	Negative	Negative
Specific gravity	1.012±0.003	1.011±0.008^NS^
Ketone bodies (mmol/L)	Negative	Negative
Bilirubin (µmol/L)	Negative	Negative
Glucose (mmol/L)	Negative	Negative

^NS^Non-significant at p<0.05. n=Number of animals, SE=Standard error

## Discussion

At present, leptospirosis is the most neglected or grossly underreported disease with a diagnostic dilemma because of its protean clinical manifestations, especially in animals. The spectrum of disease in animals ranges from mild inconsequential signs to severe form presenting with productive and reproductive problems. Moreover, only a few reports on clinical pathology of the disease in animals are available. To the best of author’s knowledge, there is the paucity of reports on clinical, pathological data on leptospirosis in goats.

### Hematology

In this study, a decrease in values of PCV, Hb, and MCHC was noted with a significant difference, while TLC revealed comparatively higher value in seropositive than seronegative goats. The present findings supported the observations of earlier workers in induced leptospirosis in Wistar rats [6]. On the same line, neutrophilia and leukocytosis in dogs infected with leptospires have been reported [7]. Similarly, neutrophilia, leukocytosis, and decreased MCHC in equine leptospirosis [5] and increased level of PCV, TLC, and decreased the value of Hb in a dog infected with *Leptospira* organism have been recorded [4]. However, the minor variations recorded could be due to certain unrelated minor pathological conditions such as anemia or day to day variation in physiological status of the animal such as estrus, pregnancy, age variation, and stress.

### Biochemical analyses

Biochemical profile, as used by many clinicians to know functional status of major vital organs, is one of the important tools which aid in revealing presumptive diagnosis and pathogenesis of the disease. In this study, the biochemical profile among seropositive goats revealed increased activity/level of ALT, AST, and total bilirubin and decreased the level of TP at a significant level (p<0.01) in comparison with seronegative goats.

Similarly, increased levels of total bilirubin, ALT, and AST in bovine [12]; increased ALP, ALT, urea, and creatinine in dogs [13]; increased ALP, ALT, total bilirubin, direct bilirubin, and creatinine in equine [5,14]; increased ALP, ALT, urea, and creatinine in Wistar rats [6]; increased BUN, creatinine, cholesterol, ALT, AST, and bilirubin in goats [3] have been reported in different studies in past. Contrary to this, Millar *et al*. [2] could not find any alteration in liver function in sheep.

It is reported that AST activity is non-specific, but ALT activity is a good indicator of liver damage among ruminants, and similarly, higher total bilirubin level occurs in liver damage [15]. Thus, the report of biochemical parameters studied presently in a limited way was suggestive of hepatic damage and resupported the general consensus that the hepatic damage does occur in leptospirosis. This result supported the report of Yang *et al*. [16], who noted that renal and hepatic damage used to occur in leptospirosis. However, in this study, various biochemical parameters did not support kidney damage possibly because kidneys continue to function apparently in a normal way for a long time due to the excess reserve of kidney tissue provided by nature till it reaches to the “point of no return.” Hypoproteinemia could be due to number of non-specific factors such as parasitism, low/poor protein level in feed, anemia, and hepatic ailment. The specific reason could not be ascertained in the absence of complete anamnesis of the individual animal as it would be only hypothetical to link it with leptospirosis.

### Urinalyses

Urinalyses among goats revealed no any specific changes in seropositive goats in comparison with seronegative goats in the present study. However, other workers have noted proteinuria, bilirubinuria, hematuria, pyuria, presence of granular casts and low specific gravity in clinical cases of dogs with severe leptospirosis [7,17]. Similarly, low specific gravity, proteinuria, and bilirubinuria have been reported in equine leptospirosis by Kumar *et al*. [14]. Besides, few workers have reported that glucosuria occurs in some cases likely as the result of tubular damage [18]. On the same line, Zaragoza *et al*. [19] have explained that increased excretion of low molecular weight proteins results from tubular damage in leptospirosis. Unfortunately, we could not locate the report on urine analysis among caprine leptospirosis.

### DFM

The presence of leptospires could not be detected in any of the screened urine samples. The possible reason could be excretion of a very low number of leptospires in urine and supported the observations made by Shivaraj *et al*. [20], who also could not detect any leptospires from 60 ovine samples (blood, tissue, and urine) though two urine samples were positive in polymerase chain reaction. On the other hand, in past, many workers have detected leptospires in various body fluids such as urine and vaginal secretion of cattle, sheep and goat using DFM [21-24]. DFM can serve as a standard screening test for early and rapid diagnosis of leptospirosis in urine or other body fluids but requires a skilled observer to differentiate between leptospires and other artefacts [25-27]. Contrary to this, it was mentioned that DFM has low indices of accuracy due to significant numbers of false positive and false negative results, but it is the method of choice for determining leptospires in cultures [9]. Therefore, it is not recommended as the sole diagnostic procedure for the early diagnosis of leptospirosis [28]. The success of DFM is also influenced by the number of leptospires discharged in various body fluids (urine, vaginal secretion, milk, etc.), and hence, excretion or discharge of a large number of leptospires increases the chance of success of DFM [22].

## Conclusion

Results of the present investigation indicated liver damage on the basis of increased level of ALT, AST, total bilirubin, and decreased level of TP in seropositive goats. In conclusion, reported clinicopathological alterations can be helpful in diagnosis and therapeutic management of leptospirosis in apparently healthy and ailing goats.

## Authors’ Contributions

PDV, JMP, JHP collected and analyzed samples, and interpreted the data as per research protocol. MCP, IHK, BPB contributed for conception, design and drafting of research paper. All authors read and approved the final manuscript.
